# Supplementary material for: Affective communication with patients with limited health literacy in the palliative phase of COPD or lung cancer: Analysis of video-recorded consultations in outpatient care
Source: PLoS One. 2022 Feb 10;17(2):e0263433. doi: 10.1371/journal.pone.0263433 (PMC8830703; doi:10.1371/journal.pone.0263433)
Supplement: Attachment — Submitted filename: Response to Reviewers.pdf [file pone.0263433.s001.pdf]

Dear Ms Schouten,

First of all we want to thank you very much for considering our paper for publication in PLOS ONE.

We also want to thank the editor and reviewer for their feedback on the manuscript and have edited the manuscript to address their concerns. We appreciate the time that you have taken to critically review our manuscript and the useful input that you have provided to improve our manuscript. We are happy to inform you and the reviewers that we were able to incorporate almost al of the suggestions, which have led to a number of significant changes. These changes can be found in the new version of our manuscript. Please find below a detailed reaction on all comments.

With kind regards,

On behalf of the authors,

Rebecca Otte

Editor and reviewers' comments and reaction

Editors comments:

As you will see, both reviewers found your findings interesting, but they have concerns about the (small) sample. Hence, I would ask you to pay special attention in your revision explaining the exact patient sample in more detail and be more cautious in drawing conclusions based on the presented results. I also agree that additional qualitative analyses would strengthen your manuscript considerably.

We note that you have indicated that data from this study are available upon request. PLOS only allows data to be available upon request if there are legal or ethical restrictions on sharing data publicly. For information on unacceptable data access restrictions, please see http://journals.plos.org/plosone/s/data-availability#loc-unacceptable-data-access-restrictions.

In your revised cover letter, please address the following prompts:
a) If there are ethical or legal restrictions on sharing a de-identified data set, please explain them in detail (e.g., data contain potentially identifying or sensitive patient information) and who has imposed them (e.g., an ethics committee). Please also provide contact information for a data access committee, ethics committee, or other institutional body to which data requests may be sent.

Reaction authors:

Thank you for the remarks. In our revision, we have utilised the valuable feedback from both reviewers to improve the manuscript. In our reactions we addressed the concerns of the reviewers concerning the study sample and strengthened the manuscript by adding several illustrative quotes. Our reaction on the specific comments of the reviewers can be found below.

Data from this study are available upon request due to the fact that the data contain potentially identifying or sensitive patient information. During the consultations, video recordings are made in which the healthcare provider and sometimes also the patient is visible. Sensitive patient information is shared about patient information, medical history and contact details. But first and foremost the data fall under the privacy regulation of the 'database communication in healthcare' (https://www.nivel.nl/nl/databank-communicatie-de-gezondheidszorg) in which is stated that Inspection and processing of data from the database is only permitted from Nivel, by whom have been designated for this purpose by the data controller and who have declared in writing that they will never act in violation of the privacy rights of the persons involved in the panel. The database is only accessible in the infrastructure of Nivel. The recordings may not leave the Nivel in original nor in duplicate, unless this is for the purpose of individual feedback to the healthcare provider concerned, or for (transport to and from) storage in an external (secure) archive.

Contact persons for the database communication in healthcare:

Prof. S. van Dulmen
+31 302729703
s.vandulmen@nivel.nl

Dr. J. Noordman
+31 302729770
j.noordman@nivel.nl

Reviewers comments (reviewer #1):

In the Discussion, the authors make a point of the finding that while a majority of cues were clinician-elicited, concerns were more often patient-elicited. The sample is way too small draw conclusions from their data. If only three more concerns had been clinician elicited, the percentage of clinician elicited utterances had been the same as for cues.

> *Thank you for your suggestions. We agree that the sample of this explorative study is small and these data must be interpreted with caution. This is also mentioned in the discussion, ('This study can only be generalised to a limited extent because of the low number of consultations available for observation and the broad range of consultation durations'). However, when it comes to the formulation of the results, we indeed agree that some statements could be formulated with more caution. If applicable, we have used the word 'might'. In this specific case, we think that emphasising the small study sample and presenting the percentages in the sentence itself also prevents misunderstanding.*
>
> *We have changed the sentence 'The majority of the concerns were initiated by the patient and coded as 'patient-elicited' (see Table 3)' to 'In our small sample, a little more than half of the concerns (55%) were initiated by the patient and coded as 'patient-elicited' (see Table 3 and Quote 1).' We also changed the wording in the Discussion part under 'added value of this study' from 'the majority of the concerns' to 'about half of the concerns'.*

The authors suggest that cues and concerns may be more prevalent in the palliative than in a curative phase of treatment and care and also that cues and concerns could be more prevalent among patients with limited health literacy. In evaluating the number of cues and concern per consultation, the authors compare their own findings with two other studies only, which both report lower frequencies. However, the literature includes a number of other studies applying VR-CoDES which report higher frequencies of cues and concerns. Moreover, the authors do not provide a comparison between low literacy and high literacy patients. The conclusion of the authors, that "patients with limited health literacy seem to express more cues and concerns than other patient population" cannot be drawn simply based on the reported findings.

> *There are indeed two variables when it comes to comparing the findings with other studies; the palliative phase and health literacy of patients. The population of this study consists of lung cancer and COPD patients with limited health literacy in the palliative phase. Especially patients with limited health literacy is a specific population that is mostly not included in research. In the manuscript we have compared the results of our study to those of Del Piccolo et al.[1], Finset, Heyn and Ruland[2] and Oguchi et al.[3], which all included cancer patients in their study population. However, none of these studies did specifically include patients in the palliative phase nor selected for health literacy. To the best of our knowledge there are no studies into affective communication in patients with limited health literacy using the VR-CoDES.*
>
> *To address your remark to make a comparison between patients in the curative or palliative setting and between low and high literate patients, we have expanded the discussion by including the article of Amundsen et al.[4] in which it is shown that the setting (either curative or palliative) did not influence the number of cues and concerns expressed by patients at the Oncology Outpatient Clinic at the University Hospital of North Norway. Again, this study did not include LHL patients. This leads us to speculate that the increased number of cues and concerns expressed by this specific patient population is indeed related to the health literacy of the patients, rather than to the specific phase of the disease, either curative or palliative.*

To sum up, the study reports interesting descriptive data on affective communication with low literacy palliative patients, but the data set is not large enough to warrant the conclusion drawn in the Discussion section. A qualitative analysis of the data set might provide a more interesting picture of affective communication in consultations with this particular patient group.

We agree that this explorative study warrants strong conclusions and have changed the wording of the results on several points to make clear that no strong conclusions can be made. Future research should replicate our findings.

Thank you for the suggestion of a qualitative analysis of the data set. The main goal of this study was to give a broad overview of the most notable findings when it comes to affective communication in patients with limited health literacy. This is also the reason why we chose to distinguish only five categories in responses of the health care provider, to give a clear indication of the differences, within the limits of the data set of this explorative study. However we think that it is indeed a good idea to add more qualitative data to the manuscript and have added an additional number of quotes to underpin the findings of the study and provide a clearer picture of the expressed cues, concerns and responses.

Reviewers comments (reviewer #2):

The study presents interesting data about a very specific patient population: patients with advanced lung disease, either COPD or lung cancer with low health literacy. I could imagine that it was hard work to gather the data and appreciate the author's attempt to have a closer look at some aspects of care for this vulnerable group of patients. However, I have some major problems with the paper in its current form: I did not find a good definition of 'palliative' care. In this age group, the very diagnosis of COPD probably means that a curative option does not exist (lung transplantation?). In addition, lung carcinoma in general is not necessarily a palliative situation, even in this age group. Therefore, I would like to read who had assigned patients to the group of 'palliative patients' based upon which criteria? If 'palliative' is just the opposite of 'curative' one could assume that everybody in this age group with a chronic disease, be it coronary heart disease or chronic arthritis, is a palliative case because curative options are no longer available, etc.

Thank you for your compliments and relevant critical question about the definition of 'palliative' care. In this study we have used the definition of 'palliative care' as provided by the WHO.[5] In earlier versions, the WHO definition of palliative care mainly stressed its relevance to patients not responsive to curative therapy, but in the latest version the WHO definition has been extended, including also other aspects (physical, emotional and spiritual needs of a patient).[6] When it comes to lung diseases the exact point of transition from a curative to a palliative approach is hard to define. This is also related to a subsequent comment, related to the methods. Indeed 'palliative' refers to a disease that is not amenable to a curative approach. We have adapted and extended the definition of 'palliative care' and 'palliative' in the methods.

We agree with the reviewer that the explanation for the determination of patients being in the palliative phase of their disease needs further clarification. In this process, either the local project manager or health care provider of the participating hospital (specialized nurses or medical specialists, always professionals with a medical background capable of making this assessment) assessed whether patients were in the palliative phase of their disease for both cancer or COPD. In their assessment they adhered to the definition of palliative care provided in the adapted methods.

In the manuscript, we have added information to the methods section, as presented below:

'In this study, the WHO definition of palliative care was applied[5], referring to patients facing problems associated with life-threating illness, in which 'palliative' might refer to a disease that is not amenable to a curative approach. Patients were included if they were aged ≥ 18 , diagnosed with lung cancer or chronic obstructive pulmonary disease (COPD) and in the palliative phase of their disease assessed by the participating medical professional or local project manager with a medical background, capable of making this assessment.'

The authors often compare their data to data from other patient populations, but I doubt whether this is justified. If neither patients in a palliative setting, as opposed to patients in early stages of breast cancer (Lidia paper) nor patients with low health literacy have been investigated, any difference found between data in this paper and the references cannot be attributed with certainty. Yet, I think as an observational study this paper is worth publishing, but I recommend being much more conservative in drawing conclusions based upon comparison with the existing literature.

We noticed that this comment is comparable with one of the feedback points of reviewer one. We have reviewed the discussion and conclusion in order to emphasize the exploratory design of this study and the corresponding limitations. Furthermore we agree that the comparison between the results of this study and existing literature can only be made to a limited extend, taking into account the different variables and settings (early stage breast cancer and the palliative phase).

Intro: Anticipating patients' emotional responses by healthcare providers (HCPs) being empathic, reassuring and supportive is called 'affective communication'
I'm not so sure whether 'anticipating' is the correct term here. The authors probably don't assume that hcps possess the witch power to predict whether the next statement of a patient will be a concern? The very next sentence, listing characteristics of affective listening makes clear that this is not the case. Instead, the task here is not to ignore affective utterances but to respond to them with one of the quoted communication acts.

You are indeed correct. We have changed 'anticipating' into 'addressing'.

Methods:
'Patients were also included if professionals considered them to be LHL (expert opinion).'
That's an interesting idea, however, drawing from our experience with hcp's inability to decide correctly, whether a patient needs the help of an interpreting service, the reader might wish to know how reliable such an estimation was. Why was this included in the inclusion criteria? How often did it occur?

We agree with the reviewer that the explanation for the inclusion of health care provider – identified patient should be clarified. With regards applying only the expert opinion in assessing health literacy in patients, this was a rare occurrence. However, the opinion of the health care provider did always prevail the objective assessment of LHL using the screening questions and the patient's educational level. There are several reasons why we choose to include the consideration provided by the health care provider. First of all patients often hide that they do not understand health information out of shame and could easily circumvent our assessment in the three screening questions.[7] Furthermore, LHL is not only found in people with a low educational level, but also in people with medium or high levels of education.

To clarify this in the manuscript we have changed this part of the methods section, indicating that the expert opinion prevailed the educational background and the three screening questions of LHL.

'patients were excluded from participating if they 1) were in the terminal phase of their disease' This makes me think of the definition of a palliative phase. I understand that this does not necessarily refer to the final phase of a disease, but then, palliative might refer to a disease that is rather not amenable to a curative approach. Is that the definition used?

This is linked to a previous comment about the definition of 'palliative' care. Here you are indeed right and the definition of 'palliative' as being 'not amenable to a curative approach' is most suitable in this situation. We have adopted this suggestion in the description of the definition as added in the methods section and elaborated the explanation for the determination of patients being in the palliative phase of their disease. Whether the patients were in the palliative phase of their disease for both lung cancer and COPD was assessed by the local project manager or professional of the participating hospital. These were always professionals with a medical background capable of making this assessment (specialized nurses or medical specialists), and in their determination adhered to the definition of palliative care given in the manuscript. To clarify this in the manuscript, we have added information to the method section, as presented below:

'In this study, the WHO definition of palliative care was applied,[5] in which 'palliative' might refer to a disease that is not amenable to a curative approach. Patients were included if they were aged ≥ 18, diagnosed with lung cancer or chronic obstructive pulmonary disease (COPD) and in the palliative phase of their disease assessed by the participating medical professional or local project manager with a medical background, both capable of making this assessment.'

'The cues and concerns of patients in the palliative phase with limited health literacy may be of a different order or more prevalent than in other patients in the curative phase.'
Besides a problem with phrasing ('different order'?) I wonder whether this sentence is justified. We see a mixture of two factors mentioned: low versus high or average health literacy, and curative versus palliative phase of a disease. I think that these two factors cannot be disentangled within this design – there are no data on patients in a similar phase of their disease being non-low in health literacy.

Here your concern is also shared by reviewer #1. Below the reaction on this point.

There are indeed two variables when it comes to comparing the findings with other studies; the palliative phase and health literacy of patients. The population of this study consists of lung cancer and COPD patients with limited health literacy in the palliative phase. Especially patients with limited health literacy is a specific population that is mostly not included in research. In the manuscript we have compared the results of our study to those of Del Piccolo et al.[1], Finset, Heyn and Ruland[2] and Oguchi et al.[3], which all included cancer patients in their study population. However, none of these studies did specifically include patients in the palliative phase nor selected for health literacy. To the best of our knowledge there are no studies into affective communication in patients with limited health literacy using the VR-CoDES.

To address your remark to make a comparison between patients in the curative or palliative setting and between low and high literate patients, we have expanded the discussion by

including the article of Amundsen et al.[4] in which it is shown that the setting (either curative or palliative) did not influence the number of cues and concerns expressed by patients at the Oncology Outpatient Clinic at the University Hospital of North Norway. Again, this study did not include LHL patients. This leads us to speculate that the increased number of cues and concerns expressed by this specific patient population is indeed related to the health literacy of the patients, rather than to the specific phase of the disease, either curative or palliative.

'This is relevant because the patients in this study expressed cues and concerns indicating distressing misunderstandings.' Did I miss this point? Where do the authors present data on the nature of the cues and concerns that would indicate that they are related to misunderstandings? Is this based upon the assumption that high health literacy is effective against misunderstandings? Then, this connection should be made explicit. Basically, I am not so sure, whether this is the case. Couldn't it be that hcps adjust their wording and the complexity of information to their perception of patients' health literacy in such a way that 'smarter' patients are getting more information thereby offsetting the benefit of a higher health literacy? The very next sentence in the discussion paper would rather strengthen my skeptical comment: patients refer to their fear of suffocation and that's not a misunderstanding but harsh reality.

Although the data indeed indicates that patients express cues and concerns related to misunderstandings, the authors agreed that there is not enough data to support this statement but rather focus on the diagnose-specific cues and concerns (for example Quote 6). It is tempting to suggest what could be the reason for the increased amount of cues and concerns expressed by the study population compared to the general population (those with sufficient health literacy), but we agree with earlier remarks that caution should be exercised when it comes to formulation conclusions based on an explorative study. Therefore the authors choose to focus on the evidently higher amount of cues and concerns in this specific study, rather than looking at the nature of the cues and concerns. Further qualitative research has to be done into the exact nature of the cues and concerns.

In the manuscript we addressed this comment by reviewing the section 'added value of this study' under Discussion. Here we stated that:

'Nevertheless, given that we know that patients with limited health literacy are more likely to experience difficulties in provider-patient communication,[8] it seems possible that these difficulties are reflected in the amount of expressed cues or concerns. In addition, the relatively high prevalence of cues and concerns about the fear of suffocation (Quote 6) would seem to be specific to the patient population (lung patients). Further studies, which take these difference settings and patient populations into account, will need to be undertaken.'

References

1. Del Piccolo L, Mazzi MA, Mascanzoni A, Lonardi M, De Felice M, Danzi OP, et al. Factors related to the expression of emotions by early-stage breast cancer patients. Patient education and counseling. 2019;102(10):1767-73.
2. Finset A, Heyn L, Ruland C. Patterns in clinicians' responses to patient emotion in cancer care. Patient Education and Counseling. 2013;93(1):80-5.
3. Oguchi M, Jansen J, Butow P, Colagiuri B, Divine R, Dhillon H. Measuring the impact of nurse cue-response behaviour on cancer patients' emotional cues. Patient Education and Counseling. 2011;82(2):163-8.
4. Amundsen A, Nordøy T, Lingen KE, Sørlie T, Bergvik S. Is patient behavior during consultation associated with shared decision-making? A study of patients' questions, cues and concerns in relation to observed shared decision-making in a cancer outpatient clinic. Patient education and counseling. 2018;101(3):399-405.

5. WHO. WHO definition of palliative care, 2002. Available from: https://www.who.int/health-topics/palliative-care.
6. Sepúlveda C, Marlin A, Yoshida T, Ullrich A. Palliative care: the World Health Organization's global perspective. Journal of pain and symptom management. 2002;24(2):91-6.
7. Barrett SE, Puryear JS. Health literacy: improving quality of care in primary care settings. Journal of Health Care for the Poor and Underserved. 2006;17(4):690-7.
8. Oosterveld M, Noordman J, Rademakers J. Kennisvraag. Samen beslissen in de spreekkamer. Ervaringen en behoeften van mensen met beperkte gezondheidsvaardigheden. 2019.
